# Effects of Environmental Temperature and Humidity on Milk Composition, Microbial Load, and Somatic Cells in Milk of Holstein Dairy Cows in the Northeast Regions of Iran

**DOI:** 10.3390/ani12182484

**Published:** 2022-09-19

**Authors:** Abdolhakim Toghdory, Taghi Ghoorchi, Mohammad Asadi, Mostafa Bokharaeian, Mojtaba Najafi, Jalil Ghassemi Nejad

**Affiliations:** 1 Department of Animal and Poultry Nutrition, Animal Science Faculty, Gorgan University of Agricultural Science and Natural Resources, Gorgan 49189-43464, Iran; 2 Department of Animal Science and Technology, Konkuk University, Seoul 05029, Korea

**Keywords:** temperature, humidity, season, milk composition, somatic cell, microbial load

## Abstract

**Simple Summary:**

Livestock performance is influenced by different elements due to the complex interactions between the individual animals and environmental conditions such as temperature and humidity. Hence, the influence of environmental temperature and humidity on milk production quality parameters needs to be investigated. The objective of this study is to elucidate the effect of environmental temperature and humidity on milk composition, microbial load, and somatic cells in the milk of Holstein dairy cows. Our findings reveal that, when temperature increased from 6.2 °C to 31.3 °C, the milk protein, fat, solids-not-fat (SNF), and somatic cell count (SCC) significantly decreased. In contrast, the microbial count in milk significantly increased, by approximately 13.7%. When humidity increased from 54% to 82%, the milk protein, fat, SNF, and SCC significantly increased. However, under the same increase in humidity, the microbial count in milk significantly decreased, by approximately 16.3%. The results indicate that there exists a correlation between different months of the year, temperature, and humidity of the environment, in terms of milk components and somatic cells. Our findings reveal that the optimum performance, in terms of milk composition, occurred in the first quarter of the year, while milk quality decreased as temperature increased and humidity decreased.

**Abstract:**

The present study aims to examine the relationships between temperature and humidity and milk composition, microbial load, and somatic cells in the milk of Holstein dairy cows. For this purpose, the temperature–humidity index, ambient temperature, and relative humidity data were obtained from the nearest weather stations. Production data were obtained from four dairy farms in Golestan province, Iran, collected from 2016 to 2021. The traits investigated were protein, fat, solids-not-fat (SNF), microbial load, and somatic cell count (SCC) in milk. The effects of the environmental temperature, humidity, month, and season on the milk composition, microbial load, and somatic cells were analyzed through analysis of variance. The effects of environmental temperature, humidity, month, and season on the milk composition, microbial load, and somatic cell composition were analyzed using a mixed procedure with a restricted maximum likelihood model. Although our findings revealed that there were significant differences in fat, protein, SNF, and SCC among the different months of the year (*p* < 0.01), no significant difference was observed in the total microbial count in milk. Environmental temperature presented significant impacts on fat, protein, SNF, SCC, and total microbial count within various temperature ranges (*p* < 0.01). When the temperature increased from 6.2 °C to 31.3 °C, the milk protein, fat, SNF, and somatic cell count significantly decreased, by approximately 4.09%, 5.75%, 1.31%, and 16.8%, respectively; meanwhile, the microbial count in milk significantly increased, by approximately 13.7%. Humidity showed an influence on fat, protein, non-fat solids, somatic cells, and total microbial count within different temperature ranges (*p* < 0.01). When the humidity increased from 54% to 82%, the milk protein, fat, SNF, and SCC significantly increased, by approximately 3.61%, 4.84%, 1.06%, and 10.2%, respectively; meanwhile, the microbial count in milk significantly decreased, by approximately 16.3%. The results demonstrate that there is a negative correlation between different months of the year, temperature, and the humidity of the environment, in terms of milk components and SCC. Our findings demonstrate that the optimum performance, in terms of milk composition, occurred in the first quarter of the year. As temperature increases and humidity decreases, milk quality decreases. Therefore, the adverse effects of environmental conditions on agricultural profits are not negligible, and strategies to better deal with the negative environmental effects are needed in order to improve milk quality in dairy cows.

## 1. Introduction

Cow’s milk is a valuable food for humans as it is a rich source of macro- and micro-nutrients, thus playing an important role in both nutrition and health protection [1]. Developing effective methods to improve milk composition has long been an active area of research, and continues to attract increasing interest in the worldwide dairy industry [2,3]. The levels of milk composition traits are influential factors that significantly affect product quality and yield in dairy cows [4]. In various developed countries, farmers are currently paid for milk deliveries based on the levels of fat and protein [5,6]. Therefore, milk composition has gained significance in the dairy industry, having a direct influence on the income of farmers and product processing. The dairy industry must make strategic decisions regarding optimizing agents that affect milk composition; in this way, they can better meet the ever-changing technological requirements and consumer preferences [7]. In general, factors affecting milk composition include the season [8], feeding [9], stage of lactation, milking interval, the health status of the cow [10,11], genetic factors, and other day-to-day variations [12,13]. The effects of environmental temperature and humidity on milk-related performance, fertility, and welfare have been widely studied in dairy cattle [14,15], and the effects of seasonal changes on milk composition have been discussed by several researchers [16,17,18].

It is widely accepted that the levels of milk components, similar to the physicochemical properties, can fluctuate extensively over the year [19]. Lactating animals are thought to be extremely sensitive to high temperature and high humidity since it has been widely accepted that environmental factors play an essential role in the health, growth, development, and lactation performance in lactating animals [2,3]. Under favorable environmental conditions, lactating animals can develop and produce milk normally. Conversely, adverse environmental conditions are known to affect the metabolism of the body, leading to declines in milk yield, milk composition, and quality in lactating animals [17,20]. Milk composition includes milk fat, milk protein, dry matter, and solids-not-fat, the decreases and changes in which lead to reduced milk quality [21,22]. Milk fat and protein contents are two major factors that fluctuate significantly during seasonal changes [22,23]. A previous study reported that, in dairy cows, high temperatures led to variations in milk composition [24].

On the other hand, a few studies have reported correlations among temperature and humidity and other indices in raw milk, including milk yield, somatic cell count (SCC), and milk losses, which are directly dependent on the cow’s health status [25,26]. The SCC is a major factor influencing udder health, as somatic cells are involved in protecting the mammary glands from infection, as part of the animal’s immune system [21,22]. The SCC in milk is affected by many factors, including species, management methods, level of milk production, and lactation stage, in addition to a range of individual and environmental factors [27].

Furthermore, Godden et al. [28] detailed that increasing heat and humidity amplified the pathogen load in the environment, resulting in a greater incidence of mastitis and increased microbial load. Therefore, climate change is assumed to affect the milk microbial count through the direct effect of months and climate variables, including average temperature and relative humidity, on the milk microbial ecology [29,30,31]. This increases the susceptibility of milk to microbial infection. Furthermore, the indirect effect of climate change on milk microbial count will be through the induction of heat stress in dairy cattle, which makes them more susceptible to pathogenic microbes [32,33].

Unfortunately, little is known regarding the effects of high levels of temperature and humidity on milk yield and quality in dairy farms of Iran. As milk quality and production are real challenges for dairy farmers, a successful strategy for improving cow farm management in Golestan Province must take into account environmental conditions such as high temperature and high humidity. Therefore, the aim of this study was to determine the effect of environmental temperature and humidity on milk composition, microbial load, and somatic cell count in Holstein dairy cows.

## 2. Materials and Methods

All experimental procedures involving animals were approved by the Animal Welfare and Ethics Committee of Gorgan University of Agricultural Sciences and Natural resources, Gorgan, Iran (approval number: N.T. 20/1115).

### 2.1. Animals, Nutrition and Maintenance Conditions

This study was conducted on four dairy farms in the northern region of Iran (Gorgan, Iran), characterized by a hot-summer Mediterranean climate. Production data were collected from 2016 to 2021. The cows were raised under the same management and environmental conditions and housed in an open loose barn. The loose open barn was designed with the overshot roof with a ridge exhaust, fans to move and exchange the air in summer, and winch curtains to block the cold wind in winter. A total mixed ration (TMR) was offered once a day at 09:00 AM, the composition of which tried to keep constant throughout the study; however little changes occurred according to the farm requirements. It included corn silage, alfalfa hay, concentrate mixture, soybean meal, and corn grain, in addition to mineral and energetic components. Cows were given ad libitum access to feed and water for 24 h. The TMR contained, on average, 47.3% dry matter (DM), 16.6% crude protein (CP), 4.96% ether extract (EE), 38.7% neutral detergent fiber (NDF), and 19.5% acid detergent fiber (ADF) on a DM basis. The percentages of DM, CP, and crude fiber in oat silage were 28.5%, 6.7%, 35.1%, and 30%, 7.11%, 34% for the spring and summer periods, respectively. The energy content in the diet of all cows was 1.7 Mcal net energy for lactation/kg DM with total digestible nutrients of 68.7%. TMR samples were taken monthly and stored at −20 °C until analysis. Furthermore, they were analyzed for DM, CP, and EE according to the AOAC [34] procedures. NDF and ADF contents were analyzed using the amylase-treated NDF (aNDF) method developed [35].

### 2.2. Samples and Laboratory Analysis

A total of 54,888 test-day records of milk composition, microbial load, and somatic cell count, collected from 2016 to 2021, were included in the study. SCS was calculated (NucleoCounter^®^ SCC-100™; Allerod, Denmark) by taking the logarithm of somatic cell count (SCC): SCS = log_2_(SCC/100,000) + 3. Ambient temperature and relative humidity data were collected from the nearest weather stations [36]. In the laboratory, the samples were immediately tested for total bacterial count, where eight consecutive dilutions were prepared from each sample, with the plated surface of each dilution in two plates containing standard plate count (SPC) medium being cultivated for a certain period of time. They were kept in a greenhouse for 72 h at a temperature of 32 °C, following which the colonies were counted and the number of bacteria per milliliter of the samples was determined [37]. The milk samples were evaluated, in terms of protein, fat, and non-fat solids, using a Milkoscan 134 model (Foss-Electric A/C, Hillerod, Denmark), according to the IDF (inverse document frequency) Standard 141B:1996. To calculate the THI (temperature–humidity index), ambient temperature and relative humidity were obtained from the nearest weather stations, and the following formula was used: THI = (1.8 × T + 32) − (0.55 − 0.0055 × RH) × (1.8 × T − 26), where T is the air temperature (in degree Celsius) and RH is the relative humidity (in percentage). The average and maximum THI of the 3 days preceding the milk sampling were used for statistical analysis [38].

### 2.3. Statistical Analysis

The data analysis was performed using the statistical package SAS Enterprise Guide 7.1 (SAS Institute Inc., Cary, NC, USA). Seasons were defined as follows: spring (March to May), summer (June to August), autumn (September to November), and winter (December to February). The effects of the environmental temperature, humidity, month, and season on the milk composition, microbial load, and somatic cell count were analyzed using an analysis of variance. Duncan’s test was used to separate the means when significance was indicated. The effects of environmental temperature, humidity, month, and season on the milk composition, microbial load, and somatic cell count were analyzed using a mixed procedure with a restricted maximum likelihood model. Environmental temperature, humidity, month, and season were included as fixed effects. Pearson correlations were calculated for the different measured parameters of milk composition and environmental factors. The values are presented as least-squares means and standard errors of the means, unless otherwise stated. Differences were considered significant if a probability (*p*) of < 0.05 was observed, and trends are discussed for variables with *p* ≤ 0.10.

## 3. Results

Meteorological data including the number, mean, minimum, and maximum of different variables measured during milk recording of farms in period from 2016 to 2021 are summarized in Table 1.

In the present study, the months of the year and milk’s main compositions (fat, protein, SSC, and SNF) had significant positive correlations, while the correlations between environmental temperature and milk composition indicators were significantly negative (Table 2).

### 3.1. Month

**Figure 1:**
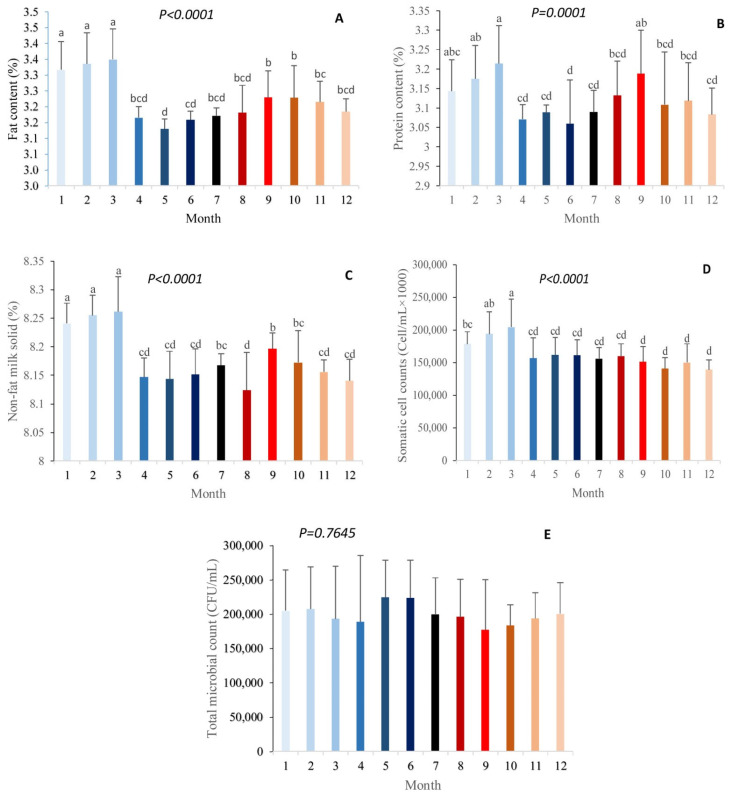
Effect of month on fat (**A**), protein (**B**), non-fat solids (**C**), somatic cell count (**D**), and total microbial count in milk (**E**).

The effects of the month on fat, protein, solids-not-fat (SNF), somatic cell count, and total microbial count in milk are shown in Figure 1. There were significant differences in fat, protein, SNF, and somatic cells among the different months of the year (*p* < 0.01), while there were no significant differences in the total microbial count in milk (*p* > 0.01). Based on our study, the percentage of milk fat was the highest in January, February, and March, while it was the lowest in May and June (*p* < 0.01). The milk fat content did not significantly vary during January, February, and March. Then, it sharply dropped (by approximately 5.52%) in April and remained steady during May, June, July, and August. The milk fat content increased by 1.24% in September, and did not significantly change during October, November, and December (Figure 1a). The percentage of milk protein was the highest in March, and the lowest in June (*p* < 0.01). The milk protein content did not change significantly in January, February, and March. Then, it decreased (by approximately 4.48%) in April and did not significantly vary during May, June, July, and August. The milk protein content increased by approximately 1.72% in September. In October, the milk protein content experienced a numerical drop of approximately 2.48%, and then remained steady during November and December (Figure 1b). The percentage of non-fat solids was the highest in January, February, and March, and lowest in August (*p* < 0.01). The content of SNF in milk was significantly similar during the months of January, February, and March; however, it was significantly reduced in April and remained steady during May and June. Then, after a numerical increase of approximately 0.19% in July, it dropped significantly by 0.53% in August. In September, the SNF content in milk was significantly raised by 0.88% and, after a numerical drop of approximately 0.20%, it did not change during November and December (Figure 1c). The somatic cell count in milk was the highest in March and the lowest in September–December (*p* < 0.01). The somatic cell count in milk was higher in February and March compared to January. Then, it remained stable from April to August and decreased and remained stable from September to December (Figure 1d). There was no significant difference in the milk total microbial count throughout the year (*p* > 0.01); however, it was numerically the highest in May and June, and lowest in September (Figure 1e).

### 3.2. Temperature

**Figure 2:**
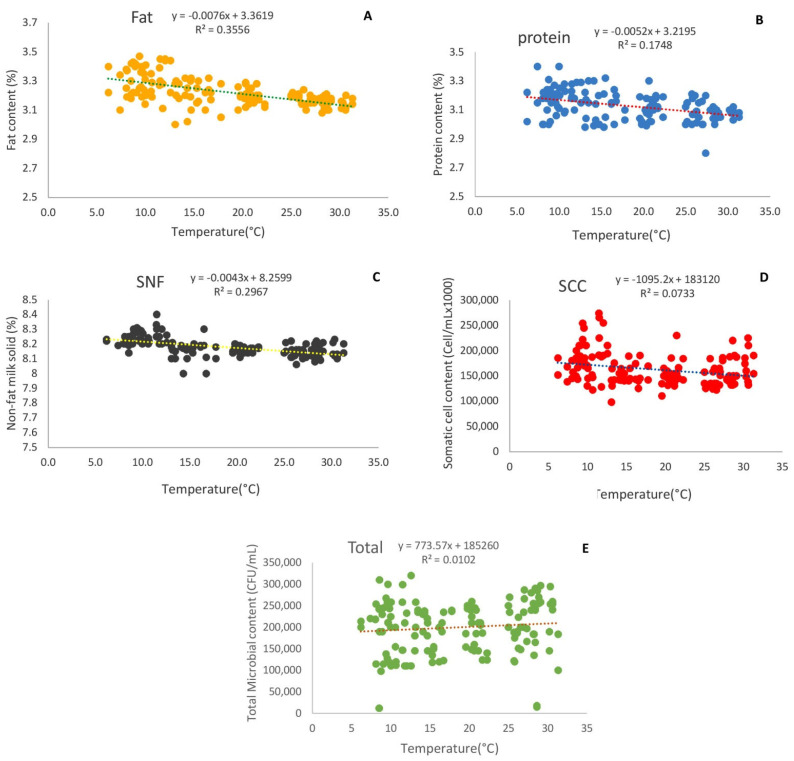
Effect of environmental temperature on fat (**A**), protein (**B**), non-fat solids (**C**), somatic cell count (**D**), and total microbial count in milk (**E**).

The effects of environmental temperature on the fat, protein, non-fat solids, somatic cell count, and total microbial count in milk are reported in Figure 2. There were significant differences in the milk fat, protein, non-fat solids, somatic cells, and total microbial count values at different temperatures (*p* < 0.01). When the temperature increased from 6.2 °C to 31.3 °C, the milk protein, fat, SNF, and somatic cell count significantly decreased by approximately 4.09%, 5.75%, 1.31%, and 16.8%, respectively (Figure 2a–d), while the total microbial count in milk significantly increased by approximately 13.7% (Figure 2e).

### 3.3. Humidity

**Figure 3:**
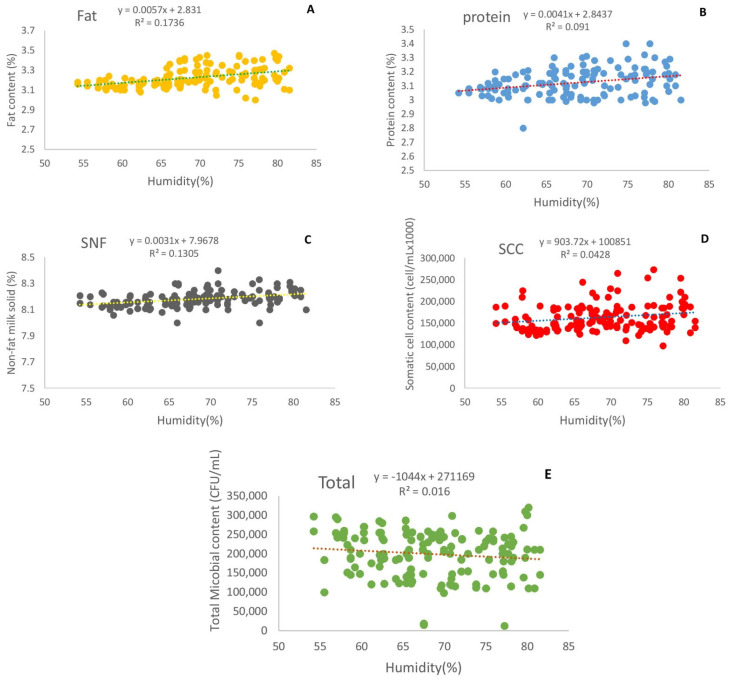
Effect of humidity on fat (**A**), protein (**B**), non-fat solids (**C**), somatic cell count (**D**), and total microbial count in milk (**E**).

The effects of the relative humidity on the fat, protein, non-fat solids, somatic cells, and total microbial count in milk are reported in Figure 3. There were significant differences in milk fat, protein, non-fat solids, somatic cell count, and total microbial count values under different relative humidities (*p* < 0.01). When the relative humidity increased from 54% to 82%, the milk protein, fat, SNF, and somatic cell count significantly increased, by approximately 3.61%, 4.84%, 1.06%, and 10.2%, respectively (Figure 3a–d), while the total microbial count in milk significantly decreased by approximately 16.3% (Figure 3e).

## 4. Discussion

It is widely accepted that reductions in milk production and milk composition are the most well-known negative responses to extreme temperature and humidity [2,39,40,41,42,43]. Environmental temperature and relative humidity have been reported to account for up to a 6% difference in the proportions of cow milk constituents [44]. The climatic conditions of the region where the animals are raised are among the most important causes of these variations [45]. In the current study, the months of the year and milk main compositions (fat, protein, SSC, and SNF) had significant positive correlations, while the correlations between environmental temperature and milk composition indicators were significantly negative. Kljajevic et al. [46] also confirmed a similar negative effect in their study for Saanen goats, with a significant negative correlation between ambient temperature and the main physicochemical characteristics of the milk. According to their report, fat was the component most highly affected by environmental temperature. They also reported that the fat content in goat milk was significantly correlated with the relative humidity (correlation coefficient of approximately 0.70). Based on the results of our study, the highest levels for the major milk composition indicators (fat, protein, SNF, and SSC) were observed in the cooler months of the year. This was also confirmed by the significant negative correlations observed between the level of milk components and temperature, as well as significant positive correlations between the level of milk composition and relative humidity. This has also been supported by the records of Barash et al. [47], who reported the highest milk yield and protein level in cows that calved in December, rather than those that calved in June. In a similar study, Zhu, et al. [48] reported that environmental conditions—particularly changes in temperature—caused decreases in milk production, fat content, protein content, dry matter, and non-fat solids in milk. Importantly, these changes were observed in July and August. Additionally, Bohmanova et al. [49] reported a sharp decline in milk components from June to August. Our results were consistent with the previous reports from April to August, showing decreases in fat, protein, and non-fat solids. Prolonged exposure to the high air temperatures over the critical physiological phase could result in decreased feed intake and a disorder of the endocrine functions of animals, indirectly affecting lactation performance and further leading to declines in milk production [2,3,50]. Another factor influencing milk yield is the photoperiod, which increases in correlation with day length [51]. Therefore, based on the latitude of farms in our study, the increase in milk yield during April, May, and June in response to the longer photoperiod could be a reason for the reduced milk fat and protein content, in terms of a dilution effect. Adverse environmental conditions may induce different levels of body stress in dairy animals, influencing the metabolism of the animal. This is further reflected in the decline in milk yield and changes in milk composition. Therefore, efforts to reduce the influence of adverse environmental conditions on dairy cows are urgently needed [48]. Appreciable evidence on the relationships between humidity and increased milk composition have been widely reported in dairy cows [26,39], while very little information exists for dairy sheep [20,24]. In general, the results from our study (Table 2) illustrate moderate negative correlation coefficients between ambient temperature and milk constituents (−0.59, −0.41, and −0.54, for fat, protein, and NFS, respectively) and a weak negative correlation (−0.27) for SSC when compared to those between relative humidity and milk constituents (0.41, 0.30, 0.36, and 0.20 for fat, protein, NFS, and SSC, respectively). We postulate, from these data, that milk composition was more susceptible to temperature than humidity in this study. In line with our results, Lim et al. [52] stated that, in summer—when the average ambient temperature (°C) and temperature–humidity index (THI) were higher—there was a negative correlation between these factors and milk production, in addition to the proportions of milk fat and protein. This was explained by Johnson [53] as greater production of heat during summer speeding up the rate of decline in milk production for cows. Additionally, the same author believed that the constant decrease in the current lactation was proportional to the length of exposure to heat stress. Bouraoui et al. [54] also attributed the reduced milk fat level to the lower intake of forage in the diet, and suggested that a total mixed ration could help to alleviate the reduction in milk fat associated with heat stress, by retaining the ratio of forage to concentrate, which ensures that the cows receive adequate fiber for normal rumen function. These results were also in accordance with the seasonal variations reported in the study of Jensen et al. [55], who observed a lower milk fat content during the summer months, as well as the study of Lindmark-Månsson et al. [56]. In addition, Hill and Wall [57] reported lower milk fat and protein contents in dairy cattle with increasing THI values. McDowell et al. [58] claimed that the decline in milk protein could occur as a result of a decrease in dry matter (energy) intake in dairy herds when the THI increases. In their review, Kadzere, Murphy, Silanikove, and Maltz [14] showed that the levels of milk protein, fat, and SNF were reduced in hot weather. Similarly, Bouraoui, Lahmar, Majdoub, Djemali, and Belyea [54] saw lower levels of milk protein and milk fat during the summer months. Lower levels of milk fat, protein, and SNF were also found in the study of Gaafar et al. [59]. Another potential postulate for the decreased fat and protein content in spring is the higher incidence of calving and, consequently, the higher number of fresh cows compared with other seasons [6]. In our study, there was a significantly negative correlation between THI and SSC, in contrast with the studies of Igono et al. [60], Zare-Tamami et al. [61], and Hammami, Bormann, M’Hamdi, Montaldo, and Gengler [26]. The main contagious pathogens initiating an increase in SCC include primary *Staphylococcus aureus* and *Streptococcus agalactiae*, as well as environmental pathogens such as *coliforms* and *Streptococcus spp.* [62]. In the present study, we found no significant correlations between the total microbial count in milk and temperature or humidity. To explain the discrepancies in the literature, it can be stated that, as high temperature intrinsically does not have any effect on the SCC in uninfected udders [63], it may be presumed that heat stress may compromise the immune status of the animals [64]. In addition, higher SCC can also be attributed to a depressed immune function due to the oxidative stress effect [26]. Furthermore, Godden, Rapnicki, Stewart, Fetrow, Johnson, Bey, and Farnsworth [28] detailed that increasing heat and humidity amplified the pathogen load in the environment, resulting in a greater incidence of mastitis and increased SSC. In the present study, no significant correlation between the milk microbial count and climate variables (i.e., month, environmental temperature, and relative humidity) was observed. However, climate change is assumed to affect the milk microbial count through the direct effect of months and climate variables, including average temperature and relative humidity, on the milk microbial ecology [29,30,31]. This increases the susceptibility of the milk to microbial infection. Furthermore, the indirect effect of climate change on the milk microbial count is expected to be mediated through the induction of heat stress in dairy cattle, making them more susceptible to pathogenic microbes [33].

## 5. Conclusions

The results presented in this paper indicate that there exists a correlation between different months of the year, temperature, and humidity of the environment, in terms of milk components and somatic cells. Our findings demonstrated that the best performance, in terms of milk composition, occurred in the first quarter of the year. As temperature increases and humidity decreases, milk quality decreases. Therefore, the effects of adverse environmental conditions on agricultural profits are not negligible, and strategies to deal with negative environmental effects should be formulated to improve dairy farm management.

## Figures and Tables

**Table 1:** Descriptive statistics of milk composition, microbial load, and somatic cell count in milk for the period 2016–2021.

Variable	Label	N	Mean	Std Dev	Minimum	Maximum
Total Protein	Protein	144	3.12	0.097	2.80	3.40
Fat	Fat	144	3.22	0.098	3.00	3.47
SNF ^1^	SNF	144	8.17	0.061	8.00	8.40
Microbial load	Total	144	199,625	59,605.7	12,000	320,000
SCC ^2^	SCC	144	162,781	31,495.4	98,000	274,000
Rainy days	Rainy days	144	7.5277778	3.8	0	18
Evaporation	Evaporation	144	111	76.1	25	274
Sunny total	Sunny total	144	196	60.1	83	333
ATR ^3^	TR	144	39.8	33.21	0	136.2
ARH ^4^	ARH	144	68.5	7.21	54.2	81.5
AT ^5^	AT	144	18.5	7.78	6.1	31.3

SNF ^1^, solids-not-fat; SCC ^2^, somatic cell count; ATR ^3^, average radiation temperature; ARH ^4^, average relative humidity; AT ^5^, average temperature.

**Table 2:** Analysis of correlation coefficients between measured parameters of milk composition and environmental factors.

Items		1	2	3	4	5	6	7	8	9	10	11	12	13	14	15	16
1	Fat	1.00															
2	Protein	0.47 ^**^	1.00														
3	SCC ^1^	0.26 ^**^	0.23 ^**^	1.00													
4	SNF ^2^	0.49 ^**^	0.37 ^**^	0.44 ^**^	1.00												
5	Microbial load	−0.12 ^ns^	−0.10 ^ns^	0.16 ^*^	−0.02 ^ns^	1.00											
6	Farm	−0.35 ^**^	−0.21 ^*^	0.004 ^ns^	0.14 ^ns^	0.05 ^ns^	1.00										
7	Month	0.46 ^**^	0.35 ^**^	0.51 ^**^	0.54 ^**^	0.01 ^ns^	0.00 ^ns^	1.00									
8	Season	0.47 ^**^	0.33 ^**^	0.50 ^**^	0.55 ^**^	0.01 ^ns^	0.00 ^ns^	0.97 ^**^	1.00								
9	Year	0.03 ^ns^	0.08 ^ns^	0.05 ^ns^	−0.07 ^ns^	−0.19 ^*^	0.00 ^ns^	0.00 ^ns^	0.00 ^ns^	1.00							
10	No. rainy day	0.27 ^**^	0.23 ^**^	0.09 ^ns^	0.21 ^**^	−0.26 ^**^	0.00 ^ns^	0.04 ^ns^	0.05 ^ns^	−0.03 ^ns^	1.00						
11	Evaporation	−0.52 ^**^	−0.38 ^**^	−0.23 ^**^	−0.46 ^**^	0.13 ^ns^	0.00 ^ns^	−0.59 ^**^	−0.62 ^**^	0.03 ^ns^	−0.55 ^**^	1.00					
12	No. sunny day	−0.45 ^**^	−0.35 ^**^	−0.25 ^**^	−0.38 ^**^	0.18 ^*^	−0.00 ^ns^	−0.49 ^**^	−0.53 ^**^	0.00 ^ns^	−0.66 ^**^	0.90 ^**^	1.00				
13	Average T ^3^	−0.59 ^**^	−0.41 ^**^	−0.27 ^**^	−0.54 ^**^	0.09 ^ns^	0.00 ^ns^	−0.62 ^**^	−0.64 ^**^	0.03 ^ns^	−0.50 ^**^	0.95 ^**^	0.84 ^**^	1.00			
14	Total rain	0.37 ^**^	0.18 ^*^	0.34 ^**^	0.36 ^**^	−0.11 ^ns^	0.00 ^ns^	0.36 ^**^	0.40 ^**^	−0.04 ^ns^	0.55 ^**^	−0.58 ^**^	−0.51 ^**^	−0.55 ^**^	1.00		
15	Average RH ^4^	0.41 ^**^	0.30 ^**^	0.20 ^*^	0.36 ^**^	−0.12 ^ns^	0.00 ^ns^	0.32 ^**^	0.34 ^**^	−0.23 ^**^	0.72 ^**^	−0.83 ^**^	−0.88 ^**^	−0.79 ^**^	0.55 ^**^	1.00	
16	THI ^5^	−0.59 ^**^	−0.42 ^**^	−0.27 ^**^	−0.55 ^**^	0.09 ^ns^	0.00 ^ns^	−0.64 ^**^	−0.66 ^**^	0.03 ^ns^	−0.49 ^**^	0.95 ^**^	0.84 ^**^	0.99 ^**^	−0.54 ^**^	−0.79 ^**^	1.00

SCC ^1^, somatic cells count. SNF ^2^, solids-not-fat. Average T ^3^, average temperature. Average RH ^4^, average relative humidity. THI ^5^, temperature–humidity index. * *p* < 0.05, ** *p* < 0.01, ^ns^ non-significant.

## Data Availability

Data are available upon a reasonable request.
